# PARP targeted Auger emitter therapy with [^125^I]PARPi-01 for triple-negative breast cancer

**DOI:** 10.1186/s13550-022-00932-9

**Published:** 2022-09-14

**Authors:** Ramya Ambur Sankaranarayanan, Alexandru Florea, Susanne Allekotte, Andreas T. J. Vogg, Jochen Maurer, Laura Schäfer, Carsten Bolm, Steven Terhorst, Arno Classen, Matthias Bauwens, Agnieszka Morgenroth, Felix M. Mottaghy

**Affiliations:** 1 Department of Nuclear Medicine, University Hospital Aachen, RWTH Aachen University, 52074 Aachen, Germany; 2 Department of Radiology and Nuclear Medicine, Maastricht University Medical Centre (MUMC+), 6229HX Maastricht, The Netherlands; 3 School for Cardiovascular Diseases (CARIM), Maastricht University, 6229HX Maastricht, The Netherlands; 4 Clinic for Gynaecology and Obstetrics, University Hospital Aachen, RWTH Aachen University, 52074 Aachen, Germany; 5 Institute of Organic Chemistry, RWTH Aachen University, 52074 Aachen, Germany; 6 Research School NUTRIM, Maastricht University, Universiteitssingel 50, 6229ER Maastricht, The Netherlands

**Keywords:** TNBC, PARP inhibitors, Targeted radionuclide therapy, Auger emitter, ^125^I, ^123^I

## Abstract

**Background:**

Triple-negative breast cancer (TNBC) lacks biomarkers for targeted therapy. Auger emitters display the best therapeutic effect, if delivered directly into the nucleus proximal to DNA. The nuclear protein Poly (ADP-ribose)-Polymerase 1 (PARP1) is a suitable target against which few inhibitors (PARPi) are clinically approved for treatment of breast cancer with germline BRCA mutation (BRCA^mut^). In this study, a theranostic approach was investigated in a TNBC xenografted mouse model by radiolabelling a close derivative of a PARPi Olaparib (termed PARPi-01) with the Auger emitters ^123/125^I.

**Methods:**

TNBC cell line MDA-MB-231 was subcutaneously implanted in female NOD/SCID mice. At a tumour size of ~ 500mm^3^, [^123^I]PARPi-01 was administered intravenously, and SPECT/CT images were obtained at 4 h or 24 h post injection (p.i). A therapy study was performed with [^125^I]PARPi-01 in 4 doses (10 MBq/dose, 10 days apart). Tumour growth was monitored by CT scans longitudinally once per week. Upon reaching study endpoint, tissues were harvested and stained with TUNEL assay for detection of apoptosis induction.

**Results:**

SPECT/CT images showed rapid hepatobiliary tracer clearance at 4 h post injection (p.i.). Retention in thyroid at 24 h p.i. suggested tracer deiodination in vivo. The tumour and liver uptake were 0.2%ID/g and 2.5%ID/g, respectively. The tumour: blood ratio was 1.3. Endogenous therapy induced a significant delay in tumour growth (doubling time increased from 8.3 to 14.2 days), but no significant survival advantage. Significantly higher apoptosis ratio was observed in [^125^I]PARPi-01 treated tumour tissues. No radiotoxicity was detected in the liver and thyroid.

**Conclusion:**

Considering the radio-cytotoxic effect in the tumour tissue and a delay on tumour doubling time, [^125^I]PARPi-01 presents a potential radiotherapeutics for treatment of TNBC. Improvements to overcome the suboptimal pharmacokinetics are necessary for its potential clinical application.

**Supplementary Information:**

The online version contains supplementary material available at 10.1186/s13550-022-00932-9.

## Background

Genomic instability is one of the established hallmarks of cancer, facilitated by the downregulation of DNA damage repair pathways, causing inability of cancer cells to repair DNA damages accurately [1, 2]. A common cause of hereditary cancers is the mutation of the DNA repair protein BRCA (BRCA^mut^). In BRCA^mut^ cells, the conventional double-strand DNA damage repair mechanism homologous recombination (HR) is deficient. Although this lack of HR is a cause of tumourigenesis, it simultaneously presents an opportunity to target these cancer cells with drugs addressing other active DNA repair mechanisms. These drugs inhibit existing DNA damage repair mechanisms, which are essential for the cell survival in absence of HR, leading the cell towards apoptosis. This phenomenon of simultaneous loss of two essential repair pathways resulting in cell death is called “Synthetic lethality” [3]. The most common target of these drugs is the nuclear DNA damage sensor protein Poly (ADP-Ribose)-Polymerase 1 (PARP1). PARP1 inhibitors (PARPi) primarily block the binding of PARP1 to the DNA damage site, thereby arresting the recruitment of repair proteins and the downstream repair process. Another mechanism of action is binding to DNA bound PARP1 and forming a PARPi-PARP1-DNA complex which blocks the release of PARP1 due to lack of recruitment of DNA repair proteins. This trapping of PARP1 stalls the replication fork causing further DNA damage [4].

Since the introduction of PARPi, clinical use of several PARPi have been approved for patients harbouring BRCA^mut^ protein, especially with ovarian and breast cancers [5]. Among breast cancer, primarily triple-negative breast cancer (TNBC) patients can benefit from PARPi. Due to the lack of expression of targetable hormone receptors (oestrogen, progesterone) or amplified human epidermal growth factor receptor (HER2) TNBC patients have only non-targeted chemotherapy or immunotherapy as standard treatment options [6]. The first approved PARPi is Olaparib for BRCA^mut^ ovarian cancer, which is prescribed at a dose of 300–400 mg twice a day [7]. In a currently published clinical study, Olaparib yielded a high response rate in treatment-naïve TNBCs revealing HR deficiency, beyond HR mutations [8]. However, due to the aggressiveness and heterogeneity of TNBC, several patients remain unresponsive to treatment with PARPi [9]. Additionally, dose limiting toxicities observed with Olaparib were anaemia, thrombocytopenia, and neutropenia [10]. Hence, there is a requirement for improvement in efficiency in PARP targeted therapy. Conjugation with radionuclides provides an option to improve the therapeutic efficiency of pharmaceuticals. Auger electron emitters (AE’s) are radionuclides that emit low energy electron clouds during their decay. AE’s exhibit a short-range and high linear energy transfer (LET) during their decay. Cytotoxicity of AE’s is deployed by multiple DNA double strand breaks induced by the intense Auger electron shower finally leading to cell apoptosis. Due to the nuclear location of PARP1, PARPi are ideal backbone molecules for radiolabelling with AE’s. ^125^I is one of the most efficient AE’s which is capable of emitting 23 Auger electrons per decay and is commercially available [11].

**Fig. 1:**
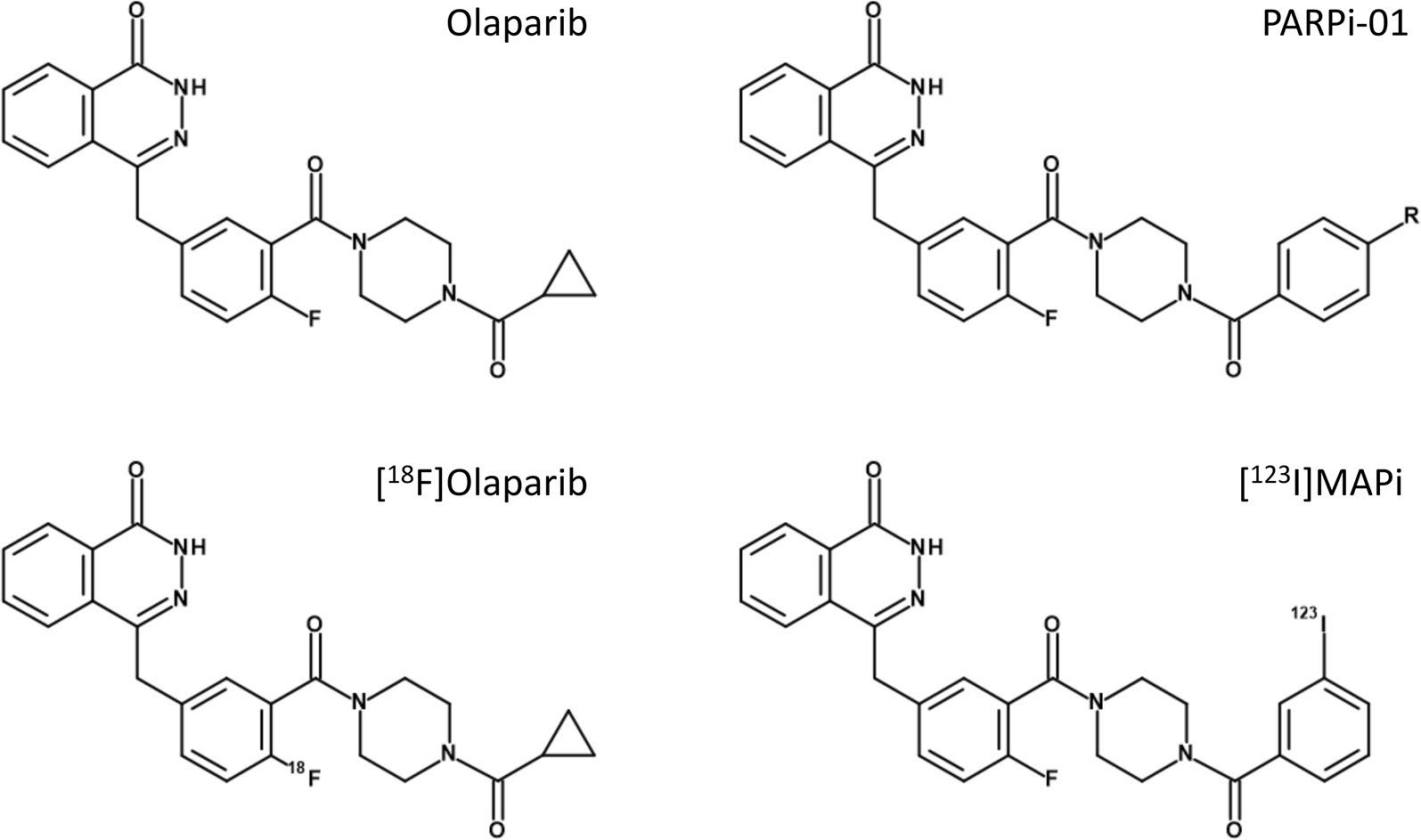
Chemical structures of PARP inhibitor Olaparib and its derivatives. The structure of the compound used in this study is PARPi-01 (R = I). The corresponding radiotracer has R = ^123/125^I. The labelling precursor used for the synthesis of [^125^I]PARPi-01 has a R = Sn(Bu)_3_. For the structure of rucaparib derivatives please refer to [41]

Use of PARPis labelled with AE’s have been recently reported for [^123^I]MAPi (Fig. 1) and [^125^I]KX1 (Rucaparib derivative) in preclinical glioblastoma and in vitro ovarian cancer models, respectively [12, 13]. The in vivo study with [^123^I]MAPi showed promising therapeutic efficacy and survival advantage upon intra-tumoural delivery in glioblastoma models. A recent study with systemic [^123^I]MAPi administration presented its high therapeutic efficacy in preclinical model of colorectal cancer with p53 deficiency [14].

Here, we have developed [^123/125^I]PARPi-01 – an inhibitor derived from Olaparib—(Fig. 1) and have analysed its potential therapeutic value in the targeted treatment of TNBC using the MDA-MB-231 model. This stem cell line shows characteristics of epithelial-to-mesenchymal transition, is representative of an aggressive metastatic TNBC [15] and hence frequently used in preclinical therapeutic studies [16]. An endogenous therapy with four cycles systematically applicated [^125^I]PARPi-01 resulted in no off-site toxicity and a delay in tumour growth despite of low tracer accumulation in the tumour.

## Materials and methods

### Radiosynthesis of [^123/125^I]PARPi-01

Radiolabelling was conducted as previously described [17] (Additional file 1: Fig. S1). Briefly, the volume of the [^123/125^I]Iodide (PerkinElmer Inc.) was reduced if necessary, by evaporation followed by redissolution in water (< 15 µL). 3 µL of a PARPi-01 precursor solution (Tributyl stannylated derivative of Olaparib, 15 mM in DCM) were evaporated to dryness, and re-dissolved in 20µL HOAc. 15 µL of the concentrated radio iodide solution were added to the acid precursor solution and labelling was started by adding 20 µL of a fresh chloramine-T solution (1 mM in MeCN). After 10 min the crude reaction mixture was purified using gradient RP HPLC with a classical C18 column (LiChrospher 100 RP18 EC 5µ, 250 × 4 mm, Merck, Germany). The product fraction was diluted with 15 mL water and was solid phase extracted by a preconditioned SepPak light C18 cartridge (WAT023501, Waters). The [^123/125^I]PARPi-01 was eluted using 1 mL CH_3_CN and evaporated to dryness. The residue was taken up in little EtOH and diluted by isotonic saline to keep the EtOH concentration below 8%. The resulting overall radiochemical yield was 78 ± 12% with purities of > 95%. Molar activity of obtained [^123^I]PARPi-01 and [^125^I]PARPi-01 were 279 ± 216 GBq/µmol (*n* = 3) and 30.5 ± 6.4 GBq/µmol (*n* = 4), respectively.

### Cell-line culture conditions

All cell lines were obtained from the Clinic for gynaecology and obstetrics, University Hospital RWTH Aachen and cultured in DMEM supplemented with 5% Fetal bovine serum and 1% Penicillin/Streptomycin. at 37 °C with 5% CO_2_. Cells in culture were tested for absence of mycoplasma every two weeks.

### Phosphorimager analysis of [^125^I]PARPi-01 nuclear uptake

Breast cancer cells were seeded (0.5 × 10^6^) in a 12-well plate and treated with [^125^I]PARPi-01 (1 MBq/10^6^ cells) for 24 h. Subcellular fractionation was performed using a Subcellular Fractionation kit (ThermoFischer inc., #78840. Protein concertation was assessed using BCA assay. SDS-PAGE was performed using the fractions (30 µg/lane) and Any KD Mini-Protean TGX gels (90 V for 1.5 h). The separated proteins were transferred to PVDF membranes using a wet blotting (60 V, 2 h) technique. The blots were then blocked with 1% Milk/TBST and incubated with a primary antibody (anti-PARP1 (1:1000); Abcam ab227244; anti-GAPDH (1:4000); cell signalling 14C10, or anti-Histon H3 1:1000) Abcam ab1791). For the phosphorimager analysis, after blotting, the blots were sandwiched with a Fujifilm Imaging plate for Bioanalyzer (BAS-MS 2040), and incubated overnight, and the protein bound activity was imaged using a phosphorimager (Typhoon FLA 7000 version 1.2). To assess the tracer uptake, both fractions were measured using a gamma counter (Wizard^2^, Perkin Elmer, USA).

### Tumour model, study design and animal care

Female NOD/SCID mice at 5–6 weeks of age (Janvier, France) was used for developing subcutaneous TNBC tumour. Animals were housed in 20–24 °C in a 12 h day light cycle. For each mouse, 3 × 10^6^ MDA-MB-231 cells were suspended in a 1:1 (volume) mixture of Culture media: Matrigel® matrix (Corning®, Product No: 356237). Mice were inoculated with this cell suspension (max 200 µl) subcutaneously in the right flank. Tumour growth was monitored daily using calliper measurements. Tumour volume was calculated daily according to the formula

$${\text{Tumour}}\;{\text{volume}} = {\text{Tumour}}\;{\text{Length}} \times \left( {{\text{Tumour}}\;{\text{breadth}}} \right)^{{2}} \times \, 0.{52}$$

Upon reaching of a tumour volume of 500 mm^3^, animals were proceeded for experiments. For biodistribution study animals were administered with [^123^I]PARPi-01 (*n* = 3). For therapy studies animals were randomized for control (*n* = 10) or [^125^I]PARPi-01 administration (*n* = 10). Randomisation was done based on equivalent distribution of tumour volumes. In addition to the daily calliper measurements image analysis of CT based tumour volumes was blinded to avoid bias. Animals were sacrificed upon completion of biodistribution study or upon reaching a humane end point for the therapy study. Humane end point is determined as reaching of either of the following conditions: 15 mm tumour diameter, 1500 mm^3^ tumour volume, or development of ulcers/ascites.

### Small animal SPECT/CT imaging

Imaging was performed with a small animal SPECT/CT system (X-Cube and γ-Cube, Molecubes, Gent, Belgium). Mice bearing subcutaneous MDA-MB-231 tumours upon reaching tumour volume of 500 mm^3^ were intravenously administered with [^123^I]PARPi-01 (9.3 ± 2.7 MBq) via lateral tail-vein using a catheter under 1.5 to 2.5% isoflurane anaesthesia in oxygen at 0.8 L/min. [^123^I]PARPi-01 was prepared by diluting the tracer in 0.9% NaCl to a total volume of 100 μL. Administered dose was calculated by subtraction of decay-corrected syringe activity post-injection from pre-injection activity. The mice were scanned either at 4 h or at 24 h post injection (p.i.). The CT acquisition settings were as follows: default high resolution protocol, 440 uA, 50 kVp, 32 ms exposure time, and 1080° rotation in a spiral mode with 960 exposures/360°; the duration of each CT scan was 5 min. A SPECT scan was initiated at the end of each CT scan. The SPECT acquisition settings were as follows: 7-headed camera equipped with a general-purpose collimator with 28 pinholes (i.e., 0.75 mm apertures and a radius of rotation of 20 mm), helical scan with 82 bed positions; the total duration of each SPECT scan was 30 min. Both CT and SPECT axial fields of view were selected at 105 mm. During the scans, the isoflurane concentration was adapted to achieve a respiratory rate between 75 and 50 breaths per minute. Images were exported and post-processed on PMOD software, version 3.8 (PMOD technologies, Switzerland).

### In vivo Biodistribution study

For biodistribution, animals imaged at 4 h p.i. were killed 24 h p.i., while the ones scanned 24 h p.i. were killed 5 days p.i.. Subsequently, organs were excised, weighed, and assayed for radioactivity in a gamma counter. Mean tumour and organ uptake was determined from decay-corrected tissue radioactivity normalized to injected dose and tissue sample weight (%ID/g tissue wet weight).

### Endogenous therapy study

Mice were administered intravenously via lateral tail vain with Saline (0.9% NaCl) or [^125^I]PARPi-01 (8.15 ± 2.9 MBq/dose) in four doses at 10-day intervals. The tumour volume was measured using [^18^F]FDG based PET/CT (Trifoil, USA) once per week. [^18^F]FDG (2.3 ± 0.4 MBq, AAA, Düsseldorf, Germany) was intraperitoneally administered and scanned 30 min later. Upon reaching 520 mm^3^ tumour volume, one additional CT imaging per week (Trifoil, USA) apart from the PET/CT was performed for monitoring tumour growth.

### Small animal PET/CT imaging

After injection with [^18^F]FDG, the mice were placed on the scanner bed and the CT scan was initiated. The exposure settings used were as follows: 130 uA, 75 kVp, 230 ms exposure time, and 360° rotation with 512 views; the duration of the CT scans was ~ 5 min. A dynamic 30 min PET scan was initiated at the end of the CT scan. The CT had an axial field of view of 91.1 mm and a PET of 112 mm. During the scans, the isoflurane concentration was adapted to achieve a respiratory rate between 75–50 breaths per minute. Mice were maintained at 1% Isoflurane and heated at 37 °C from anaesthesia induction to the end of imaging. The CT images were reconstructed using a Feldkamp filtered back projection reconstruction process to a voxel size of 0.154 × 0.154 × 0.154 mm in a 592 × 592 × 560 matrix. Using vendor software, the CT values were converted into Hounsfield units (HU) using the following formula

$${\text{HU}} = {1}000 \times \left( {\left( {\mu {\text{t }} - \, \mu {\text{w}}} \right) \, \div \, \mu {\text{w}}} \right)$$

where µw is the linear attenuation coefficient of the water and µt is the linear attenuation coefficient of the tissue. The PET data were reconstructed using a 3D ordered-subset expectation maximization (i.e., OSEM-3D with three iterations and eight subsets) with a maximum a posteriori probability algorithm (30 iterations) into a 240 × 240 × 192 image matrix (resulting in final voxel dimensions of 0.25 × 0.25 × 0.597 mm). PET normalization, CT attenuation correction, and CT scatter correction were applied to all PET reconstructions. The PET images were automatically aligned to the CT using a custom-made transformation in PMOD software package from a capillary phantom.

### Apoptosis (TUNEL) staining

Organs excised and retrieved from control (*n* = 3) and therapy mice (*n* = 3) were fixed with 4% PFA overnight at 4 °C. For cryopreservation, the organs were kept for 2–3 days at 4 °C in 30% sucrose Cryosections (6 µm) of excised and preserved organs were made using a cryostat (Leica, CM3050S). For TUNEL fluorescence quantification, therapy mice (*n* = 3) and control mice (*n* = 3) slices were stained simultaneously. The TUNEL staining kit (Roche # 11684795910) was used and protocol was followed as mentioned in the staining kit. Tissues were washed with PBS and permeabilised with 0.1 % Triton-X in 0.1 % Sodium nitrate on ice for 2 min. Following permeabilization, sections were incubated with TUNEL reaction mixture (Fluorescence labelled Terminal deoxynucleotidyl transferase enzyme dissolved in nucleotide buffer) for 1 h at 37 °C. After PBS wash, the cells were mounted with RotiMount® Vectashield containing DAPI and analysed using fluorescence microscopy (Zen Lite, Carl Zeiss). The images were processed and quantified using CellProfiler 3.0 software.

### Anti-PARP1 staining

For anti-PARP1 staining, cryosections were washed with PBS and then antigen-retrieval was performed by heating the sections in citrate buffer (pH 6) at 600 W for 20 min. The sections were washed with PBS (2 min) and then incubated with blocking buffer (1 % BSA) for 1 h. The tissues were then incubated overnight with a 1:3000 dilution of the primary antibody (Mouse Anti-PARP1; Sigma #AMAB90959). Tissues were then briefly washed with PBS and then incubated with secondary antibody (Goat Anti-Mouse DyLight 488, Invitrogen #35502) in a dilution of 1:500 for 1 h. After PBS wash, the cells were mounted with RotiMount® Vectashield containing DAPI and analysed using fluorescence microscopy (Zen Lite, Carl Zeiss).

### H&E staining

For Haematoxylin–Eosin staining the (6 µm) cryosections were washed with PBS thrice 10 min each followed by Haematoxylin (10 min) and washed with water. Subsequently tissues were incubated with Eosin (1 min) followed by wash twice in PBS for 5 min. Dehydration steps (70% Ethanol, 96% Ethanol and 100% Ethanol, Xylol) were then performed 2 min in each solvent. The tissues were then mounted and proceeded for bright light microscopy (Zen Lite, Carl Zeiss).

### Statistical analyses

All statistical analyses were performed using the Graph-Pad Prism software (Version 9.1.1). Biodistribution data is expressed as mean ± standard deviation. Kaplan–Meier Survival curve was analysed using survival analysis (Log rank Mantel-Cox test). The data points of the normalized tumour volume as a function of the time of the groups (Control, ^125^I-PARPi, Control—excluded ulcers, ^125^I-PARPi—excluded ulcers) were subjected to nonlinear regression analysis using the exponential growth formula *Y* = *Y*_0_*exp(k*x) (Malthusian). Accordingly, the doubling time is equal to ln(2)/*k*. The Goodness-of-Fit was evaluated determining R^2^ for these groups, which was 0.79, 0.81, 0.94 and 0.87, respectively, demonstrating that the selection of the model is appropriate. During regression analysis, the best-fit value k was compared between two datasets each using the extra sum-of-squares F test, testing the null hypothesis that k is the same for both datasets. Time activity analysis was performed using nonlinear exponential decay curve fitting and area under the curve was computed using the Graph-Pad Prism software. The area under the curve is expressed as total peak area ± standard error. For statistical analysis of fluorescent signals, unpaired *t*-test was used.

## Results

### Biodistribution of [^123^I]PARPi-01 in TNBC xenografted mice model

**Fig. 2:**
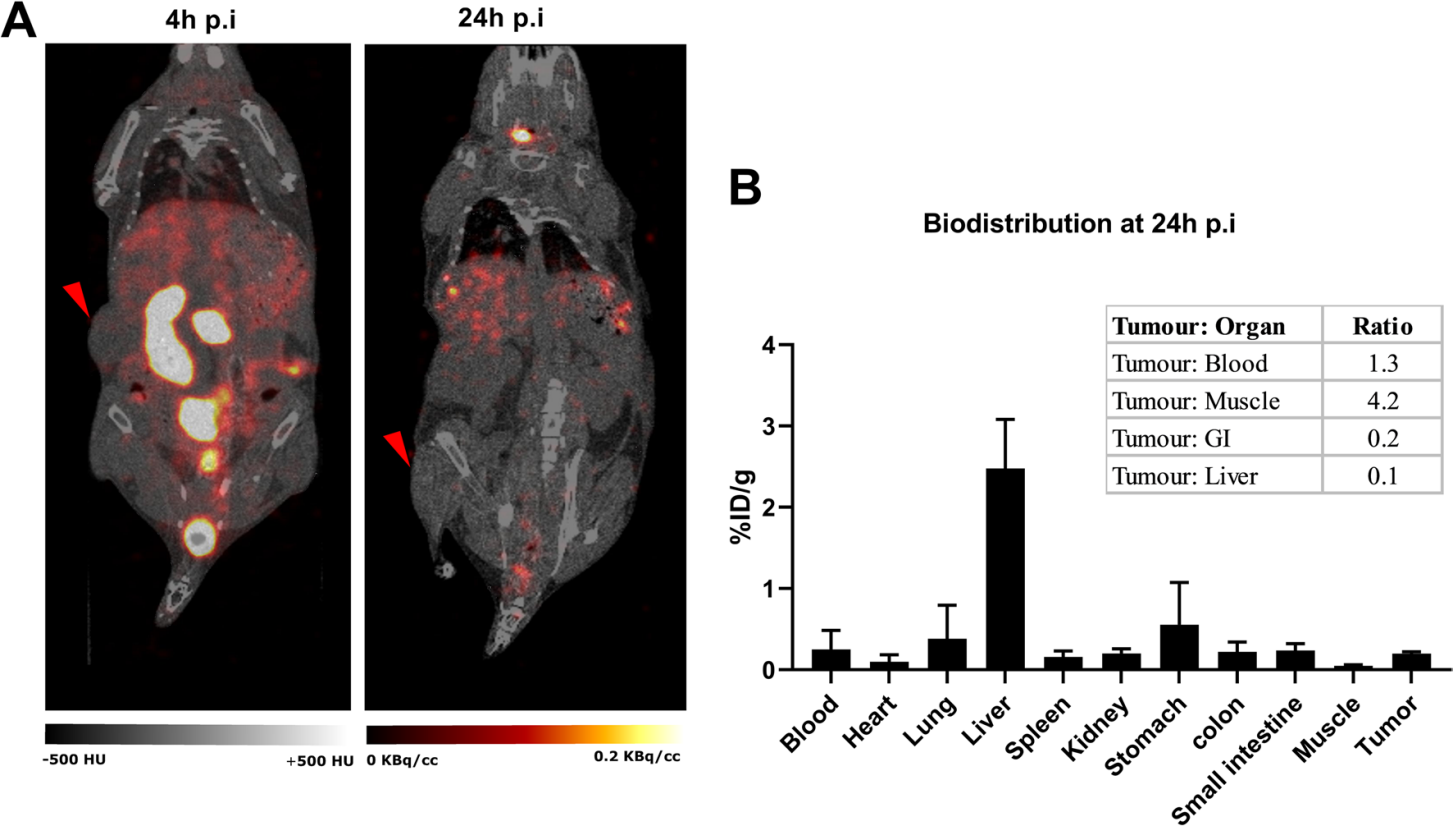
**A** Representative SPECT/CT images of biodistribution obtained at 4 h (left) and 24 h (right) post injection. Red arrow shows tumour location. **B** Ex vivo biodistribution of [^123^I]PARPi-01 at 24 h p.i in NOD/SCID mice bearing- subcutaneous MDA-MB-231 xenograft (*n* = 3)

[^123^I]PARPi-01 was injected intravenously in NOD/SCID mice bearing subcutaneous MDA-MB-231 xenografts (*n* = 3). Additional file 1: Fig. S1A shows the scheme of the [^123^I]PARPi-01 synthesis. SPECT/CT images obtained at 4 h p.i. show predominant tracer uptake in liver, the gastro-intestinal (GI) tract (small intestine, colon, and stomach) and thyroid (Fig. 2A). At 24 h p.i., tracer retention was observed only in liver and thyroid. The preferential hepatobiliary excretion of PARP-targeting tracers was already described in earlier studies [18, 19]. The high thyroid uptake and retention at 24 h p.i. is due to the dehalogenation of the tracer in vivo*.*

At 24 h p.i., animals were sacrificed and the ex vivo biodistribution data was additionally evaluated by a gamma counter analysis. However, for animals sacrificed at 5 d p.i. no quantifiable activity was measured. The results corrected for physical decay of the isotope showed that generally most of the detected activity was accumulated in liver (2.49 ± 0.6%ID/g) followed by other organs such as stomach, colon, small intestine, and kidney. This is possibly due to the high lipophilicity of the tracer. The accumulation in tumour was 0.20 ± 0.02%ID/g (Fig. 2B). The SPECT/CT image visualizes hepatic clearance as the main excretion pathway and thereby suggests that the remaining % of injected activity is cleared. The uptake ratio of tumour to blood and tumour to muscle were at 1.3 and 4.2, respectively. This indicates an almost similar tracer retention in the tumour and the blood pool, and low tracer accumulation in normal non-extracting tissues (Fig. 2B).The prolonged retention in the blood is most probably due to tracer binding with plasma proteins, as frequently indicated for lipophilic drugs [20]. The encouraging tumour: muscle ratio of 1.3 indicates a tumour targeted PARP1 mediated uptake.

### Endogenous therapy with [^125^I]PARPi-01

**Fig. 3:**
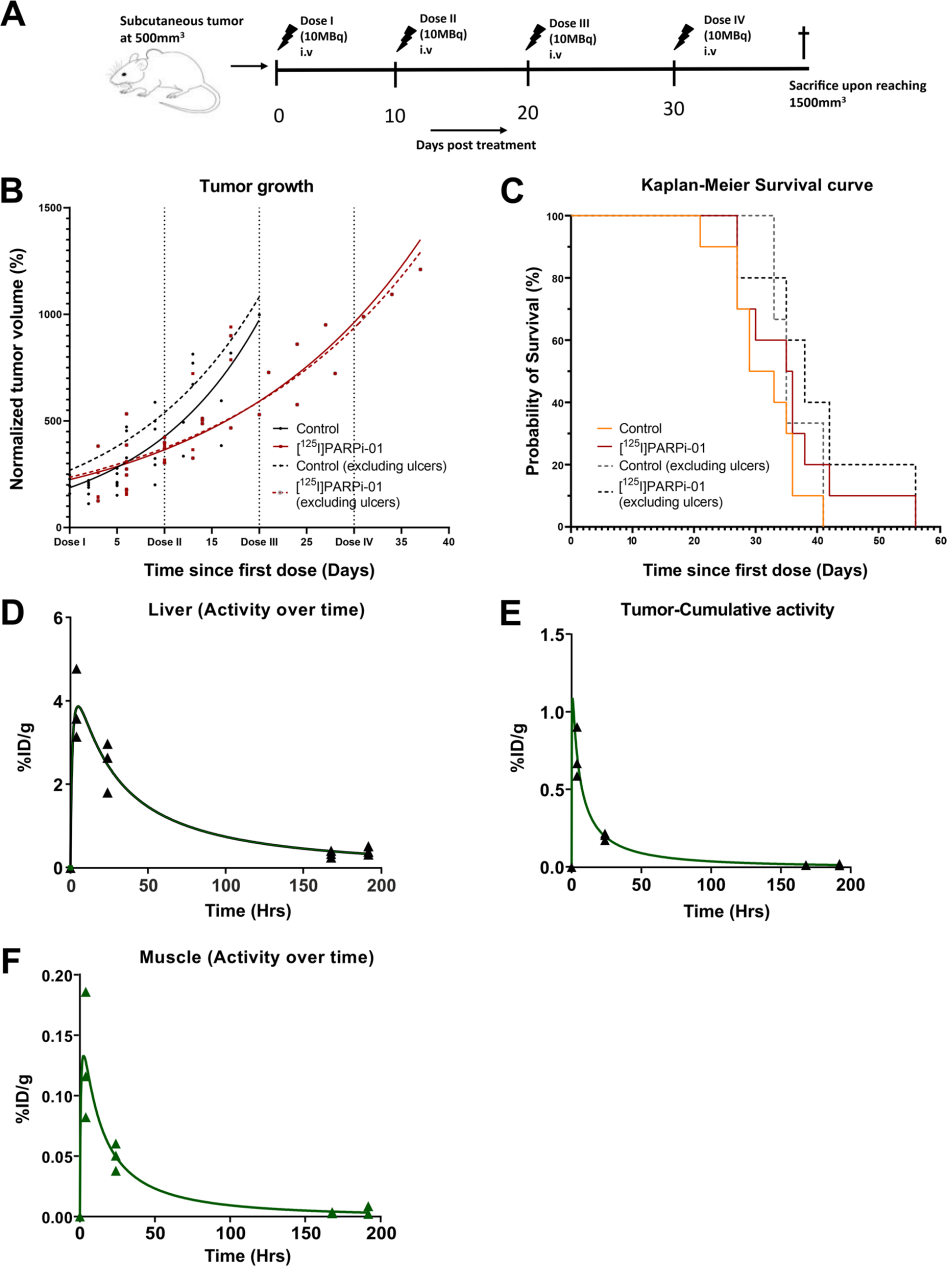
**A** Scheme of the four cycles therapy with [^125^I]PARPi-01 in TNBC xenografted NOD/SCID mice. **B** Percentage tumour growth over time shows a delay upon treatment with [^125^I]PARPi-01. **C** Kaplan–Meier survival curve of mice during the therapeutic study with mice administered with either 0.9% Saline or [^125^I]PARPi-01 in 4 doses (10 days apart; control: *n* = 10; therapy: *n* = 10). Dashed lines show survival curve that excludes animals that were eliminated due to ulcer formation (control: *n* = 3; therapy: *n* = 5) **D** Percentage of Injected dose per gram tissue (%ID/g) over time for liver** E**. for tumour and** F**. for muscle

Auger emitter-based therapeutic effect was assessed upon application of [^125^I]PARPi-01 in 4 doses (8.15 ± 2.96 MBq/dose) with 10 days between each dose in subcutaneous MDA-MB-231 models (Fig. 3A). The fractionated therapy was designed based on two recent publications [13, 21]. In the study by *Riad *et al*.,* a good tumour response without severe toxicity in normal tissue after application of 30 MBq ^125^I-labeled PARPi was shown. The same was demonstrated in the study by *Miran *et al*.,* with a fractionated therapy of 10 MBq Auger emitting nucleoside analogue following three applications with an interval of one week in an MDA-MB-231 xenografted mice model. Furthermore we observed DNA damage upon low dose PARPi application in our *in-vitro* experiments in the MDA-MB-231 cell line [17]. [^18^F]FDG based PET image analysis did not show significant difference between control and therapy cohorts with respect to SUV values and tumour metabolic volumes (Additional file 1: Fig. S2). To avoid any handling bias in calliper measurements, only CT-image-based tumour growth was analysed. CT-image-based tumour growth analysis showed that control mice (0.9% Saline injected) had a tumour doubling time of 8.3 d (95% CI: 7.4 d to 9.9 d). A significant delay in tumour growth was observed upon [^125^I]PARPi-01 treatment showing doubling time of 14.2 d (95% CI: 12.6 d to 16.1 d) (Fig. 3B, Additional file 1: Fig. S3). Comparing the treatment group with the control (with and without exclusion of animals with ulcers) we could see a significant difference between the *k* values (*P* < 0.0001 and *P* = 0.0038, respectively) and thus also the doubling time. However, Kaplan Meier-survival curve did not show any significant survival advantage upon treatment (median survival: 31 days control vs 36 days therapy; P (Mantel-cox test) = 0.2505) (Fig. 3C). A significant portion of the mice in both the control group and the therapy group were sacrificed due to reaching humane endpoints not related to tumour size. 2 mice were sacrificed due to ascites and 10 mice were sacrificed due to ulcer formation on the tumour site. When neglecting these mice, CTbased doubling time was found to be 9.95 days (control, 95% CI 8.53–11.81 days, *n* = 3) and 15.07 days (therapy, 95% CI 13.12–17.54 days, *n* = 5). Considering only this selection of mice, median survival time was 35 and 38 for control and therapy mice, respectively. Since, as shown for ^177^Lu labelled tracer, data (SUV, concentration) calculated based on SPECT images correlate with those obtained by gamma counting [22], liver, tumour, and muscle were excised for subsequent gamma counter measurements. To obtain the time activity curve and tracer retention time in each organ, the decay corrected activity (%ID/g) from the biodistribution study, therapy study and also organ activity calculated from SPECT/CT images at 4 h and 24 h p.i. were taken together and plotted over time for liver (Fig. 3D), tumour (Fig. 3E) and muscle (Fig. 3F). Tracer uptake in liver was at a peak of 3.84%ID/g at 4 h p.i. and dropped over time to 0.411%ID/g (8d p.i.). In tumour tissue, the tracer accumulation detected at 4 h p.i. with a value of 0.7219%ID/g also decreased over time (0.2% ID/g at 24 h and 0.02% at 8 days). The peak uptake of muscle was only 0.186%ID/g at 4 h p.i. and decreased to 0.002%ID/g at 8 d p.i. The overall area under the curve (retention time) in tumour was 26.53 ± 2.35 h/g as opposed to a liver retention of 284 ± 45.02 h/g, whereas the retention time of muscle was only 5.918 ± 0.98 h/g.

### Apoptotic effect of [^125^I]PARPi-01 in vivo

**Fig. 4:**
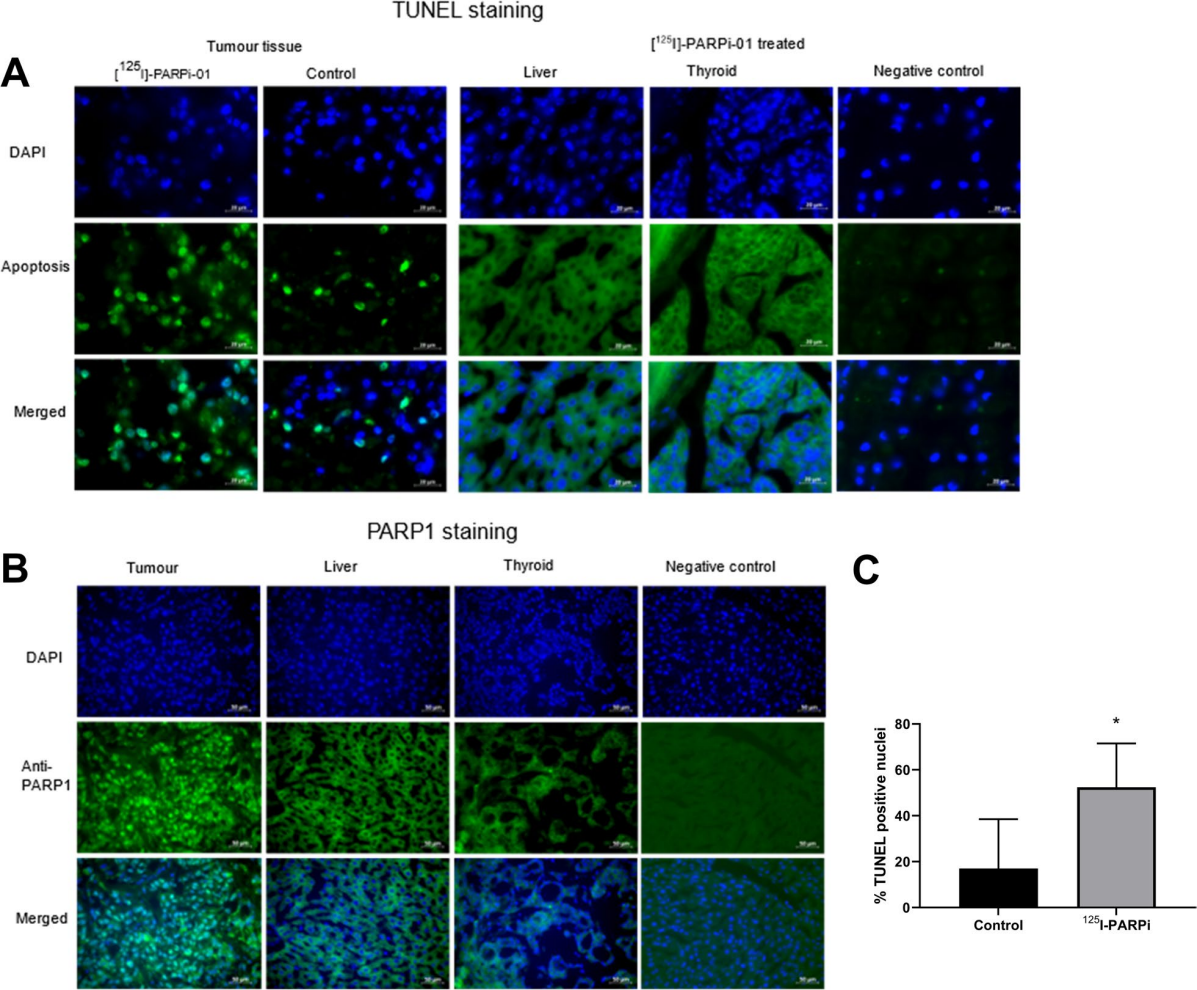
**A** TUNEL staining of 6 µm thick tumour tissues obtained post sacrifice show high apoptosis (green) in [^125^I]PARPi-01 treated tumour tissues as opposed to untreated tumour tissues. **B** Anti-PARP1 staining of target tissue (tumour) and non-target tissue (Liver and Thyroid) showed a nuclear expression of PARP1 only in tumour. Negative control without primary antibody shows no unspecific signal. **C** Quantification of percentage of apoptosis positive nuclei showed that [^125^I]PARPi-01 had significantly high (unpaired *t*-test; *p* < 0.05) apoptotic nuclei than untreated tumour tissue

In the therapy study, no animal discomfort caused due to high activity uptake was observed, possibly due to low absorbed dose in clearance organs. This no off-site radiotoxicity suggests a non-nuclear tracer localisation in liver and cells of other clearance organs. To confirm this AE induced DNA-fragmentation, apoptosis was studied by performing fluorescent terminal deoxynucleotidyl transferase mediated TUNEL staining in ex vivo tissue sections (Fig. 4A). PARP1 staining of tumour, liver and thyroid proved the nuclear localisation of PARP1 only in tumour and not in liver and thyroid. This strengthens the possibility of tumour targeted PARP1 mediated nuclear uptake of [^125^I]PARPi-01 and a non-nuclear uptake in liver and thyroid (Fig. 4B). Therefore, this contributes to the lack of apoptosis in liver and thyroid tissues as opposed to the tumour tissue. The percentage of nuclei in tumour positive for apoptosis was quantified and plotted (Fig. 4C). The number of apoptotic cells was significantly lower in tumour tissue from control mice (17.04% vs. 52,47% in therapy group, *P* < 0.05). TUNEL staining of key organs involved in the hepatobiliary excretion and in the thyroid did not show any noticeable apoptosis (Fig. 4A, Additional file 1: Fig. S4).

## Discussion

In this study the in vivo biodistribution and therapeutic effect of the Auger emitter coupled PARP inhibitor [^125^I]PARPi-01 was evaluated in a subcutaneous TNBC model. In our biodistribution study we observed a hepatobiliary clearance of [^123^I]PARPi-01, which is in line with the earlier biodistribution studies with [^18^F]Olaparib and [^123^I]MAPi [12, 18]. This results from the lipophilic nature of Olaparib with a Log P_oct_ value of 1.95, which was shown to be further elevated by a conjugation with a radionuclide (Log *P*_oct_ = 2.51) [23]. Such a lipophilic character leads in vivo to tracer binding on plasma proteins and its hepatobiliary accumulation/extraction. This explains the tumour:blood ratio of 1.3 detected at 24 h. However, high Log P might have advantageous impact on tracer biokinetics: it facilitates bypassing of bi-layered plasma membrane, protects tracer from oxidation, which increases their half-life [24]. Moreover, drugs highly bound to the plasma proteins have a low first pass-metabolism [25]. Importantly, the connection between drugs and plasma proteins is often reversible, which contributes to stable drug concentration in the blood hours after the administration [26]. Thus, considering the impact of the protein binding characteristic on biodistribution and therapy, future plasma protein binding assays with [^125^I]PARPi-01 are necessary. The SPECT/CT image visualized a strong deiodination of [^123^I]PARPi-01 in vivo as indicated by the high uptake in thyroid at 24 h p.i.. The parent compound Olaparib is known to be predominantly metabolised (84%) in liver by cytochrome P450 (CYP450), which is also known to deiodinate iodine conjugated hydrocarbons. Hence, we expect this oxidative metabolism as the cause for the observed heavy deiodination of [^123^I]PARPi-01 [27, 28]. The free [^125^I] in thyroid is not likely to present a therapeutic risk as it is expected to be localised in the cytoplasm after cellular uptake in the thyroid cells [29]. Due to the lack of PARP1 overexpression in normal cells like hepatocytes in comparison to malignant cells, tracer retention in the liver is mainly due to its hepatobiliary extraction and not due to PARP1 specific binding as detected in MDA-MB-231 cells (Additional file 1: Fig. S5). Our application of ^125^I is an optimal radioisotope choice, since ^125^I decaying in the cytoplasm has a 100-fold less radio-cytotoxic effect compared to ^125^I decaying in the nucleus, so liver damage is expected to be minimal [30].

The radiation dose to the tumour in our model is difficult to calculate. Simulations have reported high subcellular level S values, specifically for Auger emitters such as ^99m^Tc, ^111^In and ^125^I. Recent biodistribution studies with [^123^I]MAPi in healthy athymic nude mice reported the estimated absorbed dose in liver and thyroid as 0.0044 Gy/MBq and 0.0041 Gy/MBq respectively, although the intracellular distribution of [^123^I]MAPi was not taken into consideration [14]. The MIRD S-value for ^125^I in a cell nucleus to nucleus was reported to be 6.60 × 10^−3^ Gy/(Bq-s) [31]. *Goddu *et al*.,* reported a wide range of S-value for nucleus to nucleus radiation from ^125^I ranging from 3.42 × 10^−1^ Gy/(Bq-s) to 9.52 × 10^−4^ Gy/(Bq-s), assuming a homogeneous distribution of ^125^I in the nucleus with varying nucleus diameters [30]. Thus, exact calculations are difficult as even the cellular geometry plays a crucial role in the S-Values [32]. 3D-Modelling based tumour dosimetry was computed by *Lee and coworkers* using a ^125^I labelled-PARPi (^125^I-KX1) in neuroblastoma. Based on this cubic tumour model they calculated a lowest effective therapeutic dose of [^125^I]KX1 that would be four times higher than the median toxic dose for Auger emitters [33]. However, its isotopologue ([^123^I]KX1) was reported by *Riad *et al*.,* to cause DNA damage in tumour tissue upon a single dose of 29.6 MBq in a subcutaneous ovarian cancer model [13]. Also, the [^123^I]MAPi-based study showed a therapeutic response in colorectal cancer model at 74 MBq as opposed to 296 MBq predicted by *Lee *et al*.* [14]. Due to this extensive variation in cell dose that differs between cell lines and the incomparability between different cancer models, we could not directly take the therapeutic dose prediction by *Lee *et al*.,* as the sole factor for planning dosing regimen. Thus, the here presented conjugation of ^125^I to PARPi directly binding to the DNA is expected to deliver a high dose in the nucleus. The dosing regimen applied in this study, induced a significant tumour growth delay. The tumour volume doubling time increased from 8.3 days (as observed in control mice) to 14.1 days. Additionally, the amount of apoptosis in the tumours of treated mice was threefold higher compared to tumour in control mice (17.0 ± 21.5% in control vs. 52.5 ± 19.0% in therapy group), while there was no noticeable apoptosis in the liver or the thyroid. This strengthens the indication of cytoplasmic accumulation of [^125^I] in liver and thyroid and thereby a lack of radiotoxicity. Despite the observed delay in tumour growth, the difference in evaluated survival (median survival time of 36d and 31d, for treated and control group, respectively) was not significant. This is probably due to the presence of necrotic core and the longitudinal increase in the necrotic tumour volume as the tumour grows. Similarly, a lack of significance in survival was also observed in the study with [^123^I]MAPi in colorectal cancer model. Fractionated therapy with [^123^I]MAPi showed significant tumour growth delay only in p53^−/−^ subtype, but not in a p53^wt^ model [14]. This effect of [^123^I]MAPi observed in colon cancer model suggested that the DNA damaging action of Auger emitter was causing cell-death due to the synthetic lethal background provided by p53^−/−^ status of PARP1 overexpressing tumour tissue. However, in contrast to colorectal cancer, TNBC is highly heterogenous with a wide range of genetic backgrounds. In our earlier in vitro study we reported the toxicity of [^125^I]PARPi-01 in a panel of representative TNBC cell lines showing mutations in different therapy-responding genes [17]. The in this study used MDA-MB-231 cells represent a highly aggressive metastatic cell line [34]. For this cell line we reported in the previous in vitro study a significant reduction in colony formation after treatment with [^125^I]PARPi-01, however, the level of apoptosis induction remained comparable low [17]. This is possibly because it harbours a gain of function p53 mutation (p53^R280K^), KRAS^G38A^ and BRAF^G1391T^, mutations, all contributing to the survival and proliferation of the cell [35, 36]. Thus, for therapeutic application further investigation is required to establish genetic backgrounds where [^125^I]PARPi-01 will show maximum therapeutic efficiency to predict the therapeutic effect (individualized therapy). Another limitation of this study was the tumour exulceration, which has been also reported in a previous study [37]. This made monitoring of the long-term therapy response difficult by preventing completion of 4 cycles of injections in several mice. Although MDA-MB-231 is a routinely used TNBC xenograft model, its aggressive nature was a limitation to our study. Therefore, the above-discussed lack of significant therapeutic effect is attributed, at least in part, to the necessity of early animal finalisation before they reach the benefit of the therapeutic effect, and to a too low dose reached within the tumour manifestation. The last aspect is also influenced by binding to plasma protein which leads to suboptimal biodistribution of [^125^I]PARPi-01. Nevertheless, our theranostic radiotracer ([^123/125^I]PARPi-01) has also shown promise considering the highly aggressive, metastatic, nature of the MDA-MB-231 cell-line and despite the low intra-tumoural tracer accumulation. The delay in tumour growth observed by our [^125^I]PARPi-01 therapy approach is encouraging. As visualised by Phosphor-imager with subsequent SDS/Western Blot analysis, intracellular distribution was detected in cytoplasmic as well as in the nuclear fractions (Additional file 1: Fig. S5). A parallel gamma counter analysis confirmed a high nuclear tracer localization with more than 40% and 75% (for MCF-7 and both MDA-MB cell lines, respectively) of total tracer. Moreover, considering the cellular localization regulating mechanisms of PARP-1, over the treatment time we expect further translocation of cytoplasmic retained PARP-1 associated [^125^I]-PARPi-01 to the nucleus in response to occurring ^125^I-induced DSB. Thus, enhancing the delivered dose to the tumour cells along with reducing clearance is one critical improvement that will help significantly enhance the therapeutic efficacy of [^125^I]PARPi-01. Earlier studies with [^123^I]MAPi in glioblastoma models showed promising survival advantage upon intra-tumoural injection, suggesting high dose of Auger emitter targeted directly on tumour is advantageous. Unfortunately, an intra-tumoural injection is not applicable in most patients, especially in the presence of metastasis. One potential strategy to overcome all these limitations is use of drug delivery systems that specifically target TNBC biomarkers, or by modifying the structure of [^125^I]PARPi-01 to reduce its lipophilicity (thereby hopefully reducing liver uptake). As shown for Olaparib and Talazoparib, a new formulation of the drug such as nanoencapsulation presents a promising strategy to increase its pharmacological activity [38, 39]. Another nano-medical approach which has been successfully implemented for doxorubicin is encapsulating in liposomes (Doxil®). Thus, loading of nanoparticles with [^125^I]PARPi-01 will allow to improve tracer´s biokinetics (reduction of liver uptake and protection from deiodination) and by this to enhance its tumoural delivery, which can be achieved by nanoparticles functionalisation with tumour addressing ligands. Even with an improved biodistribution and pharmacokinetics, not all patients will be able to benefit from this type of treatment. As previously observed, most responders to PARPi treatment were identified as DNA repair deficient, thus stratification of patients prior to therapy with PARPi is helpful for treating therapy responsive patients only [40]. Accordingly, for the endogenous radiotherapy with [^125^I]PARPi-01 an initial selection of patient with HR deficiency would allow to optimally define responding patients.

In conclusion, [^125^I]PARPi-01 presents a promising radiotherapeutic concept for treatment of TNBC. Despite the low intra-tumoural uptake the fractionated therapy with [^125^I]PARPi-01 significantly decelerated the tumour growth. The concomitant lack of off-site toxicity in normal tissues underlays the therapeutic potential of [^125^I]PARPi-01-based therapy. However, for clinical application further improvements regarding the tumour uptake, blood stability, and fast clearance need to be addressed. This may be done by employment of an adequate drug delivery system loaded with [^125^I]PARPi-01.

## Supplementary Information

**Additional file 1**. **Fig. S1**. Reaction scheme of the radiosynthesis of [^123/125^I]PARPi-01. **Fig S2**. [^18^F]FDG based PET/CT therapy monitoring. **Fig S3**. Tumour growth curve based on CT based images for individual mice. **Fig S4**. TUNEL and H&E staining of tissues obtained from [^125^I]PARPi-01 treated mouse. **Fig S5**. SDS-PAGE/phosphor imaging of [^125^I]PARPi-01 with corresponding WB analysis of PARP1 expression

## Data Availability

The analyses of the data supporting the conclusions of this article is(are) included within the article and the supplementary file. The raw datasets used and/or analysed during the current study are available from the corresponding author on reasonable request.

## Abbreviations

TNBC: Triple negative breast cancer
PARP1: Poly (ADP- Ribose) polymerase 1 protein
PARPi: PARP inhibitor
BRCA1: Breast cancer 1 gene
KRAS: Kirsten Rat Sarcoma Virus gene
BRAF: B-Raf protooncogene
TUNEL: TdT-mediated dUTP-biotin nick end labeling
