# Correction: PARP targeted Auger emitter therapy with [^125^I]PARPi-01 for triple-negative breast cancer

**DOI:** 10.1186/s13550-023-00961-y

**Published:** 2023-02-10

**Authors:** Ramya Ambur Sankaranarayanan, Alexandru Florea, Susanne Allekotte, Andreas T. J. Vogg, Jochen Maurer, Laura Schäfer, Carsten Bolm, Steven Terhorst, Arno Classen, Matthias Bauwens, Agnieszka Morgenroth, Felix M. Mottaghy

**Affiliations:** 1 Department of Nuclear Medicine, University Hospital Aachen, RWTH Aachen University, 52074 Aachen, Germany; 2 Department of Radiology and Nuclear Medicine, Maastricht University Medical Centre (MUMC+), 6229HX Maastricht, The Netherlands; 3 School for Cardiovascular Diseases (CARIM), Maastricht University, 6229HX Maastricht, The Netherlands; 4 Clinic for Gynaecology and Obstetrics, University Hospital Aachen, RWTH Aachen University, 52074 Aachen, Germany; 5 Institute of Organic Chemistry, RWTH Aachen University, 52074 Aachen, Germany; 6 Research School NUTRIM, Maastricht University, Universiteitssingel 50, 6229ER Maastricht, The Netherlands

**Correction to: EJNMMI Research (2022) 12:60** **https://doi.org/10.1186/s13550-022-00932-9**

Following publication of the original article, it came to the authors’ attention that the Author Contributions section was partly incorrect and that the family name of the first author had been misspelled as ‘Sankaranarayana’ (instead of ‘Sankaranarayanan’). The article has since been corrected; please refer to the now-corrected article for the correct Authors Contributions information.

