# Supplementary material for: PARP targeted Auger emitter therapy with [125I]PARPi-01 for triple-negative breast cancer
Source: EJNMMI Res. 2022 Sep 14;12:60. doi: 10.1186/s13550-022-00932-9 (PMC9474773; doi:10.1186/s13550-022-00932-9)

PARP targeted Auger emitter therapy with [^125^I]PARPi-01 for triple negative breast cancer

Ramya Ambur Sankaranarayanan^1^[,](http://orcid.org/0000-0002-1267-0293) Alexandru Florea^1,2,3^, Susanne Allekotte^1^, Andreas T.J Vogg^1^, Jochen Maurer^4^, Laura Schäfer^1^, Carsten Bolm^5^_,_ Steven Terhorst^5^, Arno Classen^5^, Matthias Bauwens^1,6,2^, Agnieszka Morgenroth^1^ and Felix M. Mottaghy^1,2,3,*^

*Correspondence: [fmottaghy@ukaachen.de](mailto:fmottaghy@ukaachen.de)

1. Department of Nuclear Medicine, University Hospital Aachen, RWTH Aachen University, 52074, Aachen, Germany.
2. Department of Radiology and Nuclear Medicine, Maastricht University Medical Centre (MUMC+), 6229HX Maastricht, The Netherlands.
3. School for Cardiovascular Diseases (CARIM), Maastricht University, 6229HX Maastricht, The Netherlands
4. Clinic for Gynaecology and Obstetrics, University Hospital Aachen, RWTH Aachen University, 52074, Aachen, Germany.
5. Institute of Organic Chemistry, RWTH Aachen University, 52074, Aachen, Germany
6. Research School NUTRIM, Maastricht University, Universiteitssingel 50, 6229ER Maastricht, The Netherlands.

**Supplementary information:**

**Fig. S1.** Radiolabelling scheme of the [^123/125^I]PARPi-01.

**Fig S2.** [^18^F]-FDG based PET/CT therapy monitoring. Representative PET/CT scans of control (A) and therapy animals (B) performed before, interim and after therapy with [^125^I]PARPi-01.


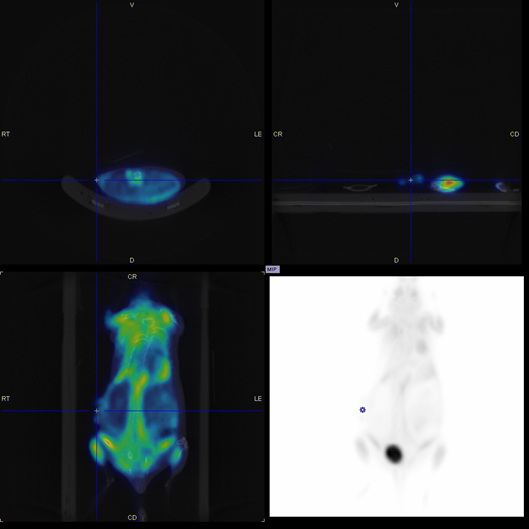

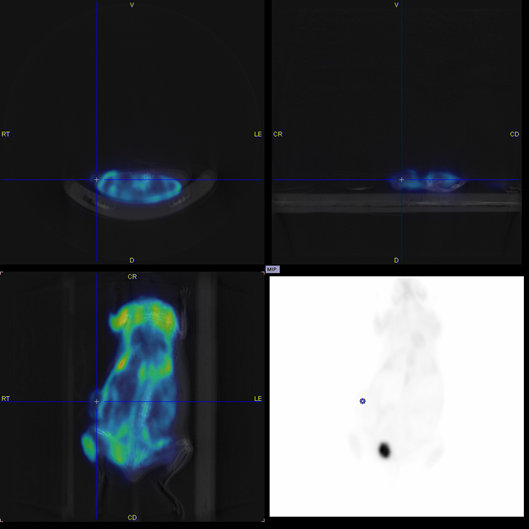


[^18^F]FDG-PET

before therapy

interim

after therapy


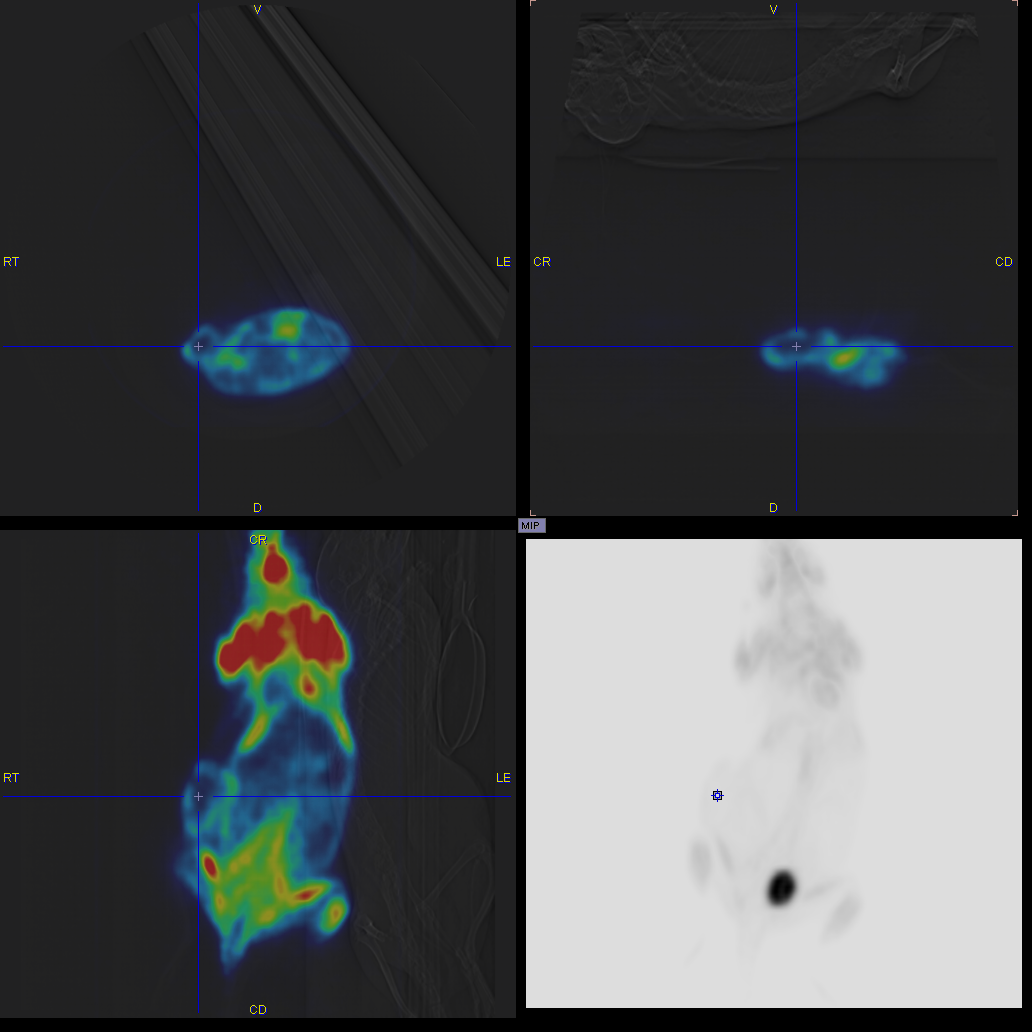

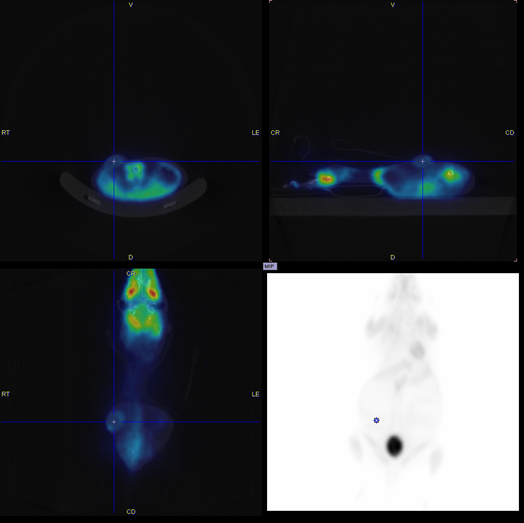

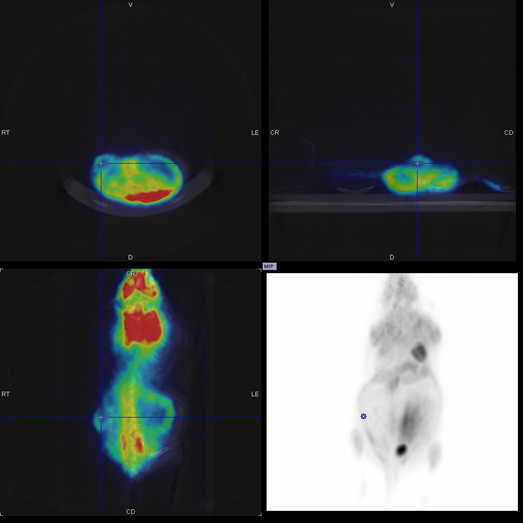

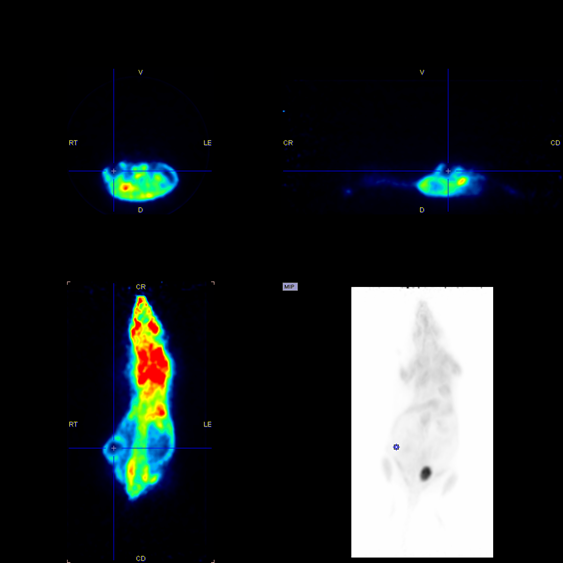


A

B


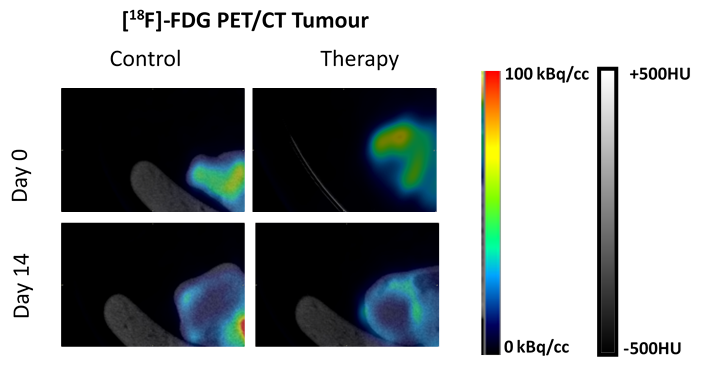


0kBq/cc

100kBq/cc

**Fig S3.** Individual tumour growth % of [^125^I]PARPi-01 treated mice are shown below. Monitoring of tumor volume was started 7 days prior to 1rst dose and then randomized blindly between therapy and control cohorts to avoid bias and hence accounts for 100% at day -7. Day 0 amounts to first dose followed by one dose every 10^th^ day.

**Fig.S4**. Representative DAPI (A) TUNEL (B) and H&E (C) staining of organs obtained from mouse treated with [^125^I]PARPi-01.


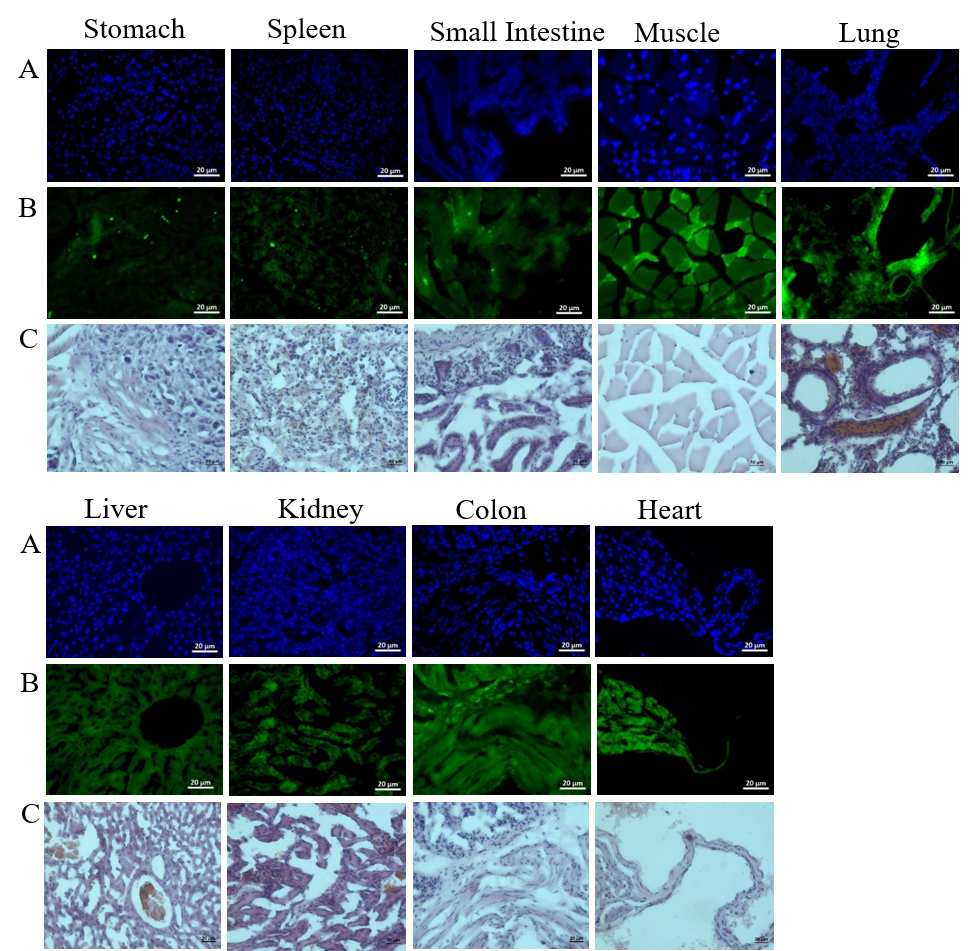


**Fig** **S5. A.** Phosphor imaging of [^125^I]PARPi-01 with corresponding **B.** Western blot analysis of PARP1 expression. (A) Cytosolic and nuclear fractions of (1) MCF7, (2) MDA-MB-231 and (3) MDA-MB-468 cells after treatment for 24h with [^125^I]PARPi-01 (1MBq/10^6^ cells) are run in an SDS page and imaged in phosphorimager for activity in PARP1 bands. (B) Corresponding Western Blots showing PARP1 expression in cytosolic and nuclear fractions with loading controls.


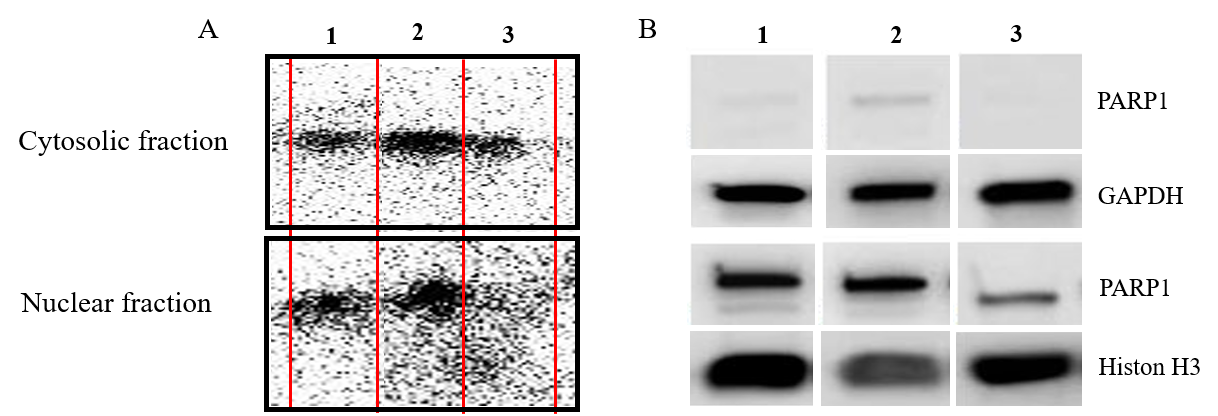

Supplement: Supplementary file 1 — Additional file 1. Fig. S1. Reaction scheme of the radiosynthesis of [123/125I]PARPi-01. Fig S2. [18F]FDG based PET/CT therapy monitoring. Fig S3. Tumour growth curve based on CT based images for individual mice. Fig S4. TUNEL and H&E staining of tissues obtained from [125I]PARPi-01 treated mouse. Fig S5. SDS-PAGE/phosphor imaging of [125I]PARPi-01 with corresponding WB analysis of PARP1 expression [file 13550_2022_932_MOESM1_ESM.docx]
